# Draft genome sequence of *Listeria monocytogenes* AZLM01 isolated from chicken sausage in Bangladesh

**DOI:** 10.1128/mra.01352-24

**Published:** 2025-05-20

**Authors:** Md. Imran Khan Masum, Zobayda Nahar, Margia Hossain Rahi, Farishta Shahel, Raidah Jahan, Hamja Hasanat, Jinath Sultana Jime, Nayeema Bulbul, Md. Fakruddin, Ashrafus Safa

**Affiliations:** 1 Department of Life Sciences, School of Environment and Life Sciences, Independent University, Bangladesh (IUB) 117116 , Dhaka, Dhaka Division, Bangladesh; 2 Department of Biochemistry and Microbiology, School of Health and Life Sciences, North South University 54495, Dhaka, Dhaka Division, Bangladesh; Wellesley College, Wellesley, Massachusetts, USA

**Keywords:** genome, food, *Listeria*

## Abstract

*Listeria monocytogenes* is a food-borne pathogen that poses a significant risk of severe infections, especially in pregnant women, the elderly, and immunocompromised individuals. This report presents the draft genome sequence of *L. monocytogenes* strain AZLM01, isolated from ready-to-eat chicken sausage—a food item that has recently gained popularity in Bangladesh.

## ANNOUNCEMENT

*Listeria monocytogenes* is a gram-positive, non-spore-forming, facultative anaerobic bacillus with low G + C content (1, 2). It is one of the most dangerous bacterial food-borne pathogens worldwide, which causes severe human diseases, such as endocarditis, hepatitis, neonatal meningitis, ophthalmitis, and joint infections (2). Although the incidence of *Listeria* infections is rare, it remains a significant and deadly food-borne disease with a hospitalization rate of over 95% (1). This study reports the draft genome sequence of *L. monocytogenes* strain AZLM01, isolated in Dhaka, Bangladesh.

The chicken sausage sample was inoculated into sterilized Buffered *Listeria* Enrichment Broth (Liofilchem, Italy), homogenized, and then enriched at 30°C for 48 h. After enrichment, the sample was subjected to serial dilution, plated onto Oxford Agar (Liofilchem, Italy), and then incubated at 35°C for 48 h. Suspected gray-black colonies with a black halo and sunken center were isolated in trypticase soy agar (Liofilchem, Italy) with yeast extract. The colonies were then identified using *L. monocytogenes*-specific primers targeting the *lmo2234* gene (forward primer: 5′-TGTCCAGTTCCATTTTTAACT-3′; reverse primer: 5′-TTGTTGTTCTGCTGTACGA-3′). A single colony of the AZLM01 strain was inoculated into 5 mL Luria-Bertani broth and incubated at 37°C for 24 h. We used the TIANamp Genomic DNA Kit (Tiangen Biotech) for DNA extraction according to the manufacturer’s instructions. A sequencing library was prepared using the Nextera DNA Flex Library Prep Kit (Illumina, San Diego, CA, USA), and the genome sequencing was conducted on the Illumina NextSeq 2000 platform with pair-end read sizes of 150 bp. A total of 10.3 M paired-end reads for strain AZLM01 were used for *de novo* genome assembly. The initial quality of the raw sequencing data was checked using FastQC version 0.11.9 (3), and the raw reads trimming and filtering were done using Trimmomatic version 0.39 (4). Assembly was performed using Unicycler version 0.4.9 (5). For genome annotation, Prokka version 1.14.6 (6) tool was used, and the Rapid Annotations Subsystems Technology server was utilized for structural gene prediction and functional annotation (7). Pangenome analysis was achieved using Roary version 3.13.0 (8). Acquired antibiotic resistance genes and virulence factors were identified using Abricate version 1.0.1 (9) and prophages by PHASTEST (10). Default parameters were used except where otherwise noted.

The final assembly for *L. monocytogenes* AZLM01 was 2,912,064 bp and was composed of 18 contigs, with an *N*_50_ value of 386,951 and an average GC content of 37.9%. Genome annotation resulted in 2,893 coding sequences, 30 tRNAs, 2 rRNAs, and 1 tmRNA. Prophage screening for the *L. monocytogenes* strain displayed three phage-related sequences of 38.8, 37.3, and 14.4 kb. The AZLM01 genome harbored antibiotic-resistant genes for lincosamide and fosfomycin antibiotics, along with 31 virulence-associated genes. The draft genome of the strain AZLM01 was compared to nine complete *L. monocytogenes* genome sequences from the NCBI database for pan-genome analysis. The pan-genome analysis identified two genes—encoding tyrosine recombinase XerC and the ATP-dependent ClpP proteolytic subunit—that are present only in the *L. monocytogenes* strain AZLM01 draft genome but absent in all the fully sequenced genome sequences from the NCBI database.

## Data Availability

The *L. monocytogenes* strain AZLM01 whole-genome shotgun project has been deposited in DDBJ/ENA/GenBank under accession no. JBJXQI000000000.1. The raw reads were deposited in the NCBI SRA database with accession number SRR31322897. The following are the accession numbers of NCBI reference sequences for *L. monocytogenes* used in pan-genome analysis: NZ_CP023752.1, NZ_CP075872.1, NZ_CP050029.1, NZ_CP168913.1, NZ_CP168923.1, NZ_LT906436.1, CP028183.1, CP023050.1, and HG421741.1.
